# Genotyping-by-Sequencing and Its Exploitation for Forage and Cool-Season Grain Legume Breeding

**DOI:** 10.3389/fpls.2017.00679

**Published:** 2017-05-09

**Authors:** Paolo Annicchiarico, Nelson Nazzicari, Yanling Wei, Luciano Pecetti, Edward C. Brummer

**Affiliations:** ^1^Centro di Ricerca per le Produzioni Foraggere e Lattiero-Casearie (FLC), CREALodi, Italy; ^2^Plant Breeding Center, Department of Plant Sciences, University of California, Davis, DavisCA, USA

**Keywords:** GBS, genetic gain, genomic selection, *Lupinus albus*, *Medicago sativa*, *Pisum sativum*, protocol, yield

## Abstract

Genotyping-by-Sequencing (GBS) may drastically reduce genotyping costs compared with single nucleotide polymorphism (SNP) array platforms. However, it may require optimization for specific crops to maximize the number of available markers. Exploiting GBS-generated markers may require optimization, too (e.g., to cope with missing data). This study aimed (i) to compare elements of GBS protocols on legume species that differ for genome size, ploidy, and breeding system, and (ii) to show successful applications and challenges of GBS data on legume species. Preliminary work on alfalfa and *Medicago truncatula* suggested the greater interest of *Ape*KI over *Pst*I:*Msp*I DNA digestion. We compared KAPA and NEB *Taq* polymerases in combination with primer extensions that were progressively more selective on restriction sites, and found greater number of polymorphic SNP loci in pea, white lupin and diploid alfalfa when adopting KAPA with a non-selective primer. This protocol displayed a slight advantage also for tetraploid alfalfa (where SNP calling requires higher read depth). KAPA offered the further advantage of more uniform amplification than NEB over fragment sizes and GC contents. The number of GBS-generated polymorphic markers exceeded 6,500 in two tetraploid alfalfa reference populations and a world collection of lupin genotypes, and 2,000 in different sets of pea or lupin recombinant inbred lines. The predictive ability of GBS-based genomic selection was influenced by the genotype missing data threshold and imputation, as well as by the genomic selection model, with the best model depending on traits and data sets. We devised a simple method for comparing phenotypic vs. genomic selection in terms of predicted yield gain per year for same evaluation costs, whose application to preliminary data for alfalfa and pea in a hypothetical selection scenario for each crop indicated a distinct advantage of genomic selection.

## Introduction

Next generation sequencing techniques provide many molecular markers at low cost by sequencing single nucleotide polymorphism (SNP) sites in a fraction of the genome. While many array-based procedures require prior knowledge of target sequences, other methods, such as complexity reduction of polymorphic sequences (CRoPS) (van Orsouw et al., 2007) and restriction enzymes site-associated genomic DNA sequencing (RAD-seq) (Baird et al., 2008), skip sequence discovery and explore SNP variation in DNA fragments cut by a restriction enzyme (RE). The use of methylation sensitive REs, which tend to avoid highly repetitive DNA regions, helps targeting restriction sites that are relatively random and evenly distributed along the genome in gene-rich regions.

In RAD-seq, restriction fragments are ligated to adapters on one end, sheared and size selected, then ligated with adapters on the other end and finally PCR amplified, before sequencing the region flanking the restriction site. Multiplexing many genotypes in a single sequencing reaction can be done using a unique barcode sequence (4 to 8 bp long) in one end of the adapters before ligation. A similar but simplified procedure termed genotyping-by-sequencing (GBS) was proposed by Elshire et al. (2011) using *ApeK*I as a “frequent cutter” RE. GBS skips the shearing and size selection stage, combining the ligation of both adapters into one step. Another improvement of GBS is the modulation of length and nucleotide composition of barcode sequences. The current GBS cost per DNA sample (inclusive of sample preparation) is on the order of 25–30 €. This represents a possible cost reduction of about 40–60% relative to SNP array-based genotyping, with greater reduction for relatively small experiments. At these costs, genomic selection (Heffner et al., 2009) can be applied even to crops of moderate or modest economic importance and/or with no sequenced genome. However, relative to array-based genotyping, GBS presents challenges in coping with missing data and their imputation, and it may require optimization for different species.

The success of GBS depends on the number of polymorphic SNP markers that can be identified. Statistically robust SNP calling depends on the number of sequencing reads per SNP, with a threshold set to 2 or 3 reads for pure lines of inbred species (where heterozygosity is absent), 6 for outbred diploids, and 11 for homozygous loci of an outbred autotetraploid species such as alfalfa, for type I error rate <5%. One way to increase the read depth across all genotypes being sequenced is to minimize the number of fragments that are able to be sequenced. Poland et al. (2012a) developed a modified GBS protocol with double enzyme digestion by *Pst*I and *Msp*I RE to reduce the number of target sites while increasing their read depth, using a common adapter that allows amplification only of fragments cut by a different RE at each end. Selected amplification may also be pursued by using primers that selectively ligate or amplify a subset of the restriction fragments while using (for example) the *ApeK*I RE (Sonah et al., 2013). GBS protocols that restrict the number of target sites produce markers with greater read depth (for a fixed total number of reads per flow cell) but do not imply necessarily more exploitable SNP markers than the original method by Elshire et al. (2011), because of the lower number of sequenced DNA fragments and, for infrequent cutting RE or RE combinations, because of greater number of large DNA fragments that are amplified less frequently thereby failing to reach the threshold read depth. Finally, the *Taq* polymerase adopted for DNA fragment amplification may change the numbers of successfully sequenced SNP markers, e.g., by using a less selective polymerase such as KAPA in place of the NEB polymerase adopted in the original method (Elshire et al., 2011).

Greater cultivation of grain and forage legumes is recognized as a key issue for making cropping systems more sustainable in terms of greenhouse gas emissions, energy consumption, soil fertility, and crop diversification (Schneider and Huyghe, 2015), as well as for reducing the marked and increasing insufficiency of feed proteins in large regions such as Europe and China (Pilorgé and Muel, 2016). The main reason for insufficient cultivation of legumes is their modest yielding ability compared with cereals (Reckling et al., 2016), which highlights the importance of exploring the potential of GBS-based genomic selection for higher yield in these crops (Pandey et al., 2016). One study on soybean confirmed the value of genome-enabled predictions by displaying accuracy close to 0.60 (Jarquín et al., 2014). While prediction of pure line performance is the obvious aim of genomic selection in inbred species, predicting the breeding value of candidate parent genotypes for synthetic varieties is the objective of greatest practical interest in outbred species such as alfalfa or other important forage legumes, e.g., white clover or red clover (Annicchiarico et al., 2015a). In a recent study, genomic selection for alfalfa breeding value for forage yield in two contrasting populations achieved an accuracy around 0.35, which could largely offset the gain per unit time from field selection based on progeny test (Annicchiarico et al., 2015b). In another study, accuracies of up to 0.40 were found using a model developed from an initial Cycle 0 population to predict biomass yield of the Cycle 1 population (Li et al., 2015). GBS data may prove valuable also in plant breeding contexts other than genomic selection, namely, in studies of genome-wide association, variety distinctness, diversity, and phylogenetic relationships. Diploid alfalfa such as *Medicago sativa* L. subsp. *caerulea*, while being less useful agronomically than tetraploid alfalfa (subsp. *sativa*), can be studied to produce genomic information of interest also for tetraploid material (Sakiroglu and Brummer, 2016).

With a focus on alfalfa and the cool-season grain legumes pea (*Pisum sativum* L.) and white lupin (*Lupinus albus* L.), this study aimed (i) to assess the effect of RE, *Taq* polymerase and primers on the amount of GBS information generated, and (ii) to report on some successful applications and challenges of genomic selection based on GBS data.

## Materials and Methods

### Experiment 1: Comparison of Restriction Enzyme × *Taq* Polymerase Combinations in *M. sativa* and *M. truncatula*

This study included 2 genotypes of tetraploid *M. sativa* (Altet4 and NECS141) described in Khu et al. (2013), 2 of diploid *M. sativa* (MS-13 and MS-186) described in Han et al. (2012), 2 of diploid *M. sativa* (CC78-68 and CF15-13) described in Li et al. (2011), and 2 reference genotypes of *M. truncatula* [A17, genome-sequenced (Young et al., 2011); and R108].

We compared the utility of two RE protocols [*ApeK*I (Elshire et al., 2011) or *Pst*I:*Msp*I (Poland et al., 2012a)] in combination with *Taq* polymerases obtained from either New England Biolabs (NEB) or Kapa Biosystems (KAPA). After DNA extraction, we prepared libraries for each of the 4 RE × *Taq* combinations (*ApeK*I and NEB, *ApeK*I and KAPA, *Pst*I:*Msp*I and NEB, *Pst*I:*Msp*I and KAPA). Libraries using *Ape*KI were generated using Elshire et al.’s (2011) protocol with minor modifications. Briefly, 100 ng of each DNA sample (quantified with a Quant-iT PicoGreen dsDNA assay kit, Life Technologies, P7589) was digested with *ApeK*I (NEB, R0643L) and then ligated to a unique barcoded adapter and a common adapter (7.0 ng of the adapter stock were used per the titration test on one alfalfa DNA sample). Equal amounts of the ligated product of each of the eight samples were pooled and cleaned up with QIAquick PCR purification kit (QIAGEN, 28104) for PCR amplification. To generate the *ApeK*I and NEB library, 50 ng template DNA was mixed with NEB 2X Taq Master Mix and 2 primers (with 5 nmoles each) in a 50 μl total volume and amplified on a thermocycler with 18 cycles of 10 s of denaturation at 98°C, 30 s of annealing at 65°C, 30 s extension at 72°C. To generate the *ApeK*I and KAPA library, the only differences were the adoption of the Kapa Library Amplification Readymix (Kapa Biosystems KK2611) instead of NEB and the use of 12 instead of 18 cycles in the amplification program. *Pst*I-*Ms*pI and NEB and *Pst*I-*Ms*pI and KAPA were generated according to the protocol by Poland et al. (2012a) with modifications. In each library, we intentionally doubled the amount of *M. truncatula* genotypes, to obtain more reads on those samples.

After de-multiplexing, we identified 64-bp long DNA fragment tags for each genotype using the Stacks pipeline (Catchen et al., 2013), and randomly extracted the same number of reads from each genotype in each library to make fair comparisons among protocols. The same number of reads were randomly extracted using the “fastq-sample” function in the “fastq-tools”^1^. Because the lowest number of useful reads for any genotype × RE × *Taq* combination was 1.3 M, we extracted 1.25 M reads from each combination to represent approximately the case of 192-plex multiplexing (since the Illumina HiSeq2000 can deliver over 240 M useful reads per lane). We further analyzed data with 2.5 M reads extracted for each genotype, to test approximately the case of 96-plex but excluded the results for tetraploid *M. sativa*, which failed to reach this threshold in all RE × *Taq* combinations. For each genotype and GBS protocol, we counted the number of tags available for minimum read thresholds of 2, 6, and 11, reporting mean values for the 3 genotype groups (*M. truncatula*; diploid *M. sativa*; tetraploid *M. sativa*).

### Experiment 2: Comparisons of KAPA vs. NEB *Taq* Polymerases for Sequencing Bias

We compared KAPA and NEB for selective amplification across DNA fragments that differed for size or for content of nitrogenous bases, comparing the tag distribution expected from *ApeK*I *in silico* digestion of the *M. truncatula* reference genome with those observed from *ApeK*I digestion with each polymerase (using the highest number of available reads, i.e., 6.6 M). For each tag generated by the two polymerases, we obtained the targeted restriction fragment by BLAST search on the Mt4.0v1 reference genome from genotype A17 downloaded from http://www.jcvi.org/cgi-bin/medicago/download.cgi. For DNA fragment size analysis, we computed for KAPA and NEB-based libraries the percentage of tags belonging to each of 15 defined size classes, and compared them with the values expected from *in silico* digestion. For bias relative to content of nitrogenous bases, we computed the percentages of fragments classified to each of seven classes defined based on the GC content of the DNA fragments and compared them with the expected values from *in silico* digestion.

### Experiment 3: Comparison of *Taq* Polymerase × Selective Primer Combinations in Pea, White Lupin, and Alfalfa

This study included the four diploid and two tetraploid genotypes of *M. sativa* described in Experiment 1, six pea genotypes, and four white lupin genotypes. The pea genotypes included three cultivars (Attika, Isard, and Kaspa) that were parents of three connected inbred line populations (Annicchiarico et al., 2017). For each cultivar, we extracted two random genotypes obtained from different commercial seed lots. Although presumably identical, genotyping data revealed genetic differences between the two genotypes of each cultivar in all cases. The lupin germplasm included one genotype from the French cultivar Lucky, and three landrace genotypes that were selected on the basis of their genetic diversity in a prior study performed on a wider genotype set. The source populations of these genotypes were the landraces La568 from Algeria, La646 from the Canary Islands, and LAP123 from Italy, described in the world collection study by Annicchiarico et al. (2010).

The *ApeK*I RE was used for all protocols as described earlier. We compared the 12 protocols represented by KAPA or NEB polymerases in combination with the non-selective primer proposed in the original method (Elshire et al., 2011) or one of 5 3′ primers we designed that had 5 to 8 specific bases in order to selectively amplify fragments. All primers were synthesized by Eurofins MWG Operon.

We used the UNEAK pipeline (Lu et al., 2013) for SNP discovery and genotype calling. For fair comparisons, we randomly identified 1.5 M reads from each genotype × protocol combination in each library as described earlier. A higher number would have resulted in one or more genotypes being dropped from the analysis. For each protocol and genotype combination, we assessed the number of polymorphic SNPs (as 64-bp long sequences with one polymorphism) that were shared by all test genotypes, setting a minimum read depth of 3 for pea and white lupin (inbred species), 6 for diploid alfalfa, and 11 for tetraploid alfalfa. For tetraploid alfalfa, which featured more total reads per genotype, we repeated the assessment for a scenario of 2.5 M total reads per genotype.

### Number of Polymorphic Markers and Predictive Ability of Genomic Selection Models in Different Data Sets

This part of the study summarized the number of polymorphic markers and the predictive ability of genomic selection based on cross-validations for yield or quality traits in data sets of tetraploid alfalfa, pea or white lupin. The main sources of data were provided by alfalfa studies in Annicchiarico et al. (2015b) and Biazzi et al. (2017) and the pea study in Annicchiarico et al. (2017), where genotyping protocols, phenotypic procedures for production traits, SNP calling procedures and details of other bioinformatic analyses are reported. Most analyses were performed using various packages of the R software. Some findings from these studies were recalled here to summarize the impact on genomic selection predictive ability of different thresholds for genotype missing data, marker imputation method, and genomic selection model. An initial filtering step excluded markers with a minor allele frequency below 2.5%.

The study by Annicchiarico et al. (2015b) described the genotyping of 154 parent genotypes from a broadly based reference population including Mediterranean germplasm (Me population) and 124 parent genotypes from a broadly based reference population comprising germplasm from the Po Valley, northern Italy (PV population). These germplasm sets were phenotyped separately for forage yield on the basis of densely grown half-sib progenies issued by polycrossing in isolation each set of parents (as convenient for genome-enabled prediction of breeding values: Annicchiarico et al., 2015a). The GBS protocol included *ApeK*I as RE in both populations, while using NEB and KAPA polymerases for PV and Me, respectively. SNP genotype calling distinguished only three classes, namely, the two homozygous ones (AAAA or aaaa), and the heterozygous one (pooling the variants Aaaa, AAaa, and AAAa). A filtering step removed heterozygous loci with less than 4 aligned reads, and homozygous loci with less than 11 reads (thereby reducing the probability to falsely call AAAa or Aaaa heterozygotes as a homozygote to 4.22%).

Biazzi et al.’s (2017) study focused on the same set of half-sib progenies of the Me population, and assessed various quality traits of stems and leaves across three growing conditions (summer harvest, full irrigation; summer harvest, suspended irrigation; autumn harvest). We currently added original genomic selection information on two quality traits, namely, protein content and digestibility of NDF, that were assessed on pooled leaf and stem foliage of the material. For each trait, we compared five genomic selection models, namely, Ridge Regression BLUP, Bayes A, Bayes B, Bayes C, and Bayesian Lasso (Gianola, 2013), for predictive ability based on cross-validations as described in Biazzi et al. (2017) for quality traits of stems and leaves, using a threshold of 30% for missing genotype SNP data and missing data imputation by the *K*-Nearest Neighbor method.

In the pea study (Annicchiarico et al., 2017), 315 F_6_ recombinant inbred lines (RILs) belonging to three populations derived by connected crosses between Attika, Isard, and Kaspa were assessed for grain yield under severe terminal drought stress under a field rainout shelter. The GBS protocol included *ApeK*I as RE and KAPA as *Taq* polymerase. We currently anticipated unpublished genomic selection results for prediction of grain yield in a 3-replicate field experiment carried out in Lodi (northern Italy) under organic farming conditions and autumn sowing in the season 2013–2014. We held a minimum read depth of 4 for SNP genotype calling, because the F_6_ generation contained some heterozygous loci. We assessed results for a minimum read depth of 6 as well, obtaining less SNP markers but very similar predictive ability (data not reported), as observed already for grain yield under severe drought (Annicchiarico et al., 2017). Genomic predictions were based on the Ridge Regression BLUP model, 30% threshold for missing genotype SNP data, and missing data imputation by the *K*-Nearest Neighbor method. The model was trained on all populations joined in a single data set and took account of population structure, as performed already in Annicchiarico et al. (2017).

In this paper, predictive ability, i.e., the correlation between genome-based predicted values and observed values, was used as an estimate of prediction accuracy, i.e., the correlation between genome-based predicted values and true breeding values. In several studies on inbred crops, prediction accuracy was estimated by dividing prediction ability by the square root of the broad-sense heritability on a line mean basis, thereby obtaining a higher value that accounts for possible experiment errors in the estimation of breeding values. This correction, however, may introduce a bias when cross-validations are applied to data of the same experiment (Lorenz et al., 2011), as in the current case. We preferred to adopt cautiously lower estimates of prediction accuracy, to minimize the risk of overoptimistic results for genomic selection.

We reported the number of polymorphic markers also for two data sets of white lupin. The former included 288 genotypes sorted out from the world landrace collection in Annicchiarico et al. (2010). The genotypes belonged to seven major historical cropping regions (Madeira-Canaries, Portugal, Spain, Maghreb, Egypt, East Africa, Near East), from each of which we sampled 8 to 10 landrace populations, and 3 or 4 genotypes per landrace population. The latter lupin set included 191 RILs issued by the cross between the cultivar Kiev Mutant and the Ethiopian landrace P27174 [which were used for constructing the first linkage map of this species: Phan et al. (2007)]. RIL DNA samples were obtained from the Institute for Plant Genetics in Poznan in the framework of a joint research work with CREA. GBS protocols for lupin data sets were identical to those for pea that were described in Annicchiarico et al. (2017).

We reported, as a reference, also numbers of polymorphic markers and/or genomic selection predictive ability from other published studies on forage or cool-season grain legumes. The information from the study by Li et al. (2015) was relative to a genomic selection model for forage yield constructed on clonally phenotyped plants when applied to selected intercrossed material evaluated in a further selection stage in the same location. We averaged the results across two target locations (one in NY State and one in Québec) as this scenario, implying predictions essentially for additive genetic effects, can be compared to prediction of breeding values in the other reported data sets.

Finally, we briefly compared GBS-based genomic selection vs. phenotypic selection in terms of predicted yield gains per unit time in relation to hypothetical selection scenarios and rough estimates of selection costs. Cost estimates (which were inclusive of DNA extraction for GBS) were based on our own experience, recent quotes from genomic platforms, and feedback on phenotyping costs provided by various colleagues and breeding programs, realizing, of course, that GBS costs may decrease in the near future.

## Results

### Comparison of Restriction Enzyme × *Taq* Polymerase Combinations in *M. sativa* and *M. truncatula*

**FIGURE 1:**
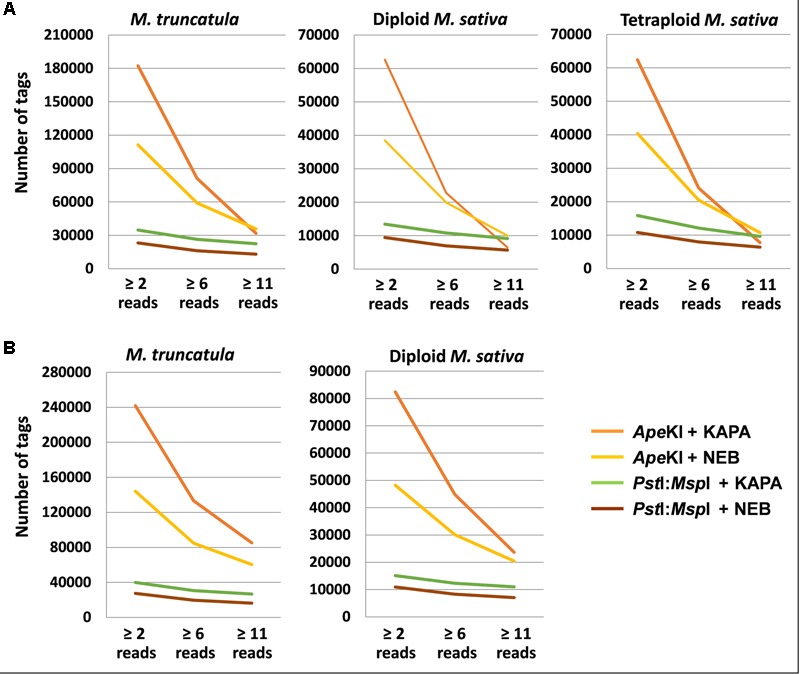
**Mean number of tags of two restriction enzyme (RE) × 2 *Taq* polymerase combinations for three read depths, in three sets of genotypes of *Medicago truncatula* or *M. sativa*. (A)** 1.25 M total reads per genotype; **(B)** 2.5 M total reads per genotype.

For the scenario of 1.25 M total reads per genotype, the combination of *ApeK*I and KAPA provided a higher number of tags than the other GBS protocols in all sets of genotypes, for minimum read depths of 2 reads per tag (useful for pure lines of inbreds such as *M. truncatula*) or 6 (useful for the outbred diploid *M. sativa* subsp. *sativa*) (**Figure 1A**). However, the four protocols exhibited few differences and a slight advantage for *ApeK*I and NEB, when requiring at least 11 reads per tag (as necessary to identify homozygotes for tetraploid alfalfa) (**Figure 1A**). The responses of diploid and tetraploid alfalfa were nearly identical (**Figure 1A**), as expected since their 1C genomes are the same size and we held read depth constant across genotypes.

The shift from 1.25 to 2.5 M total reads per genotype improved the relative performance of *ApeK*I-based GBS at higher read depths, particularly when combined with KAPA, whereas *Pst*I:*Msp*I produced fewer tags than *ApeK*I across all relevant read depths (**Figure 1B**). Under this scenario, *ApeK*I and KAPA were top-ranking for number of tags even at minimum read depth of 11 (**Figure 1B**). Results of diploid alfalfa for this read depth are likely to apply also to tetraploid alfalfa under 2.5 M total reads per genotype, when considering the high consistency of results between diploid and tetraploid alfalfa verified under the 1.25 M total read scenario.

### Comparisons of KAPA vs. NEB *Taq* Polymerases for Sequencing Bias

**FIGURE 2:**
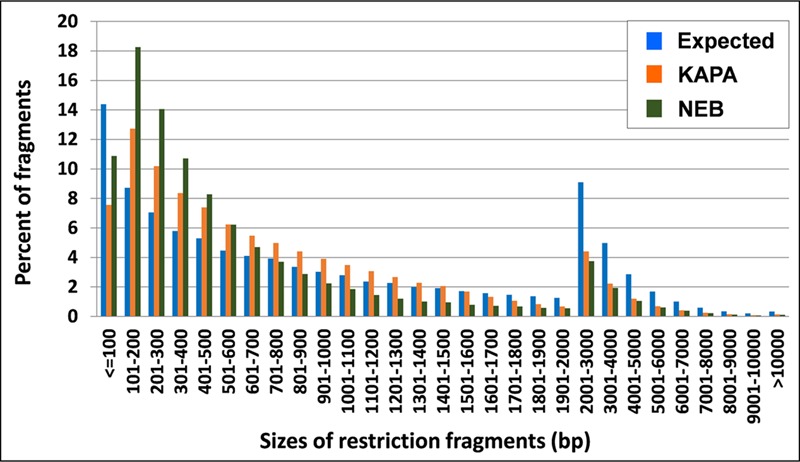
**Tag distribution across different sizes of DNA fragments in *M. truncatula* as expected from *Ape*KI *in silico* digestion and observed from 2 *Taq* polymerases**.

KAPA provided more uniform amplification than NEB over fragment sizes of *M. truncatula*, on the basis of smaller differences between observed and expected tag frequencies for this polymerase. In particular, NEB distinctly overamplified fragments in the range of 100–500 bp (**Figure 2**), i.e., those that make the greatest contribution to GBS tags (Sonah et al., 2013).

**FIGURE 3:**
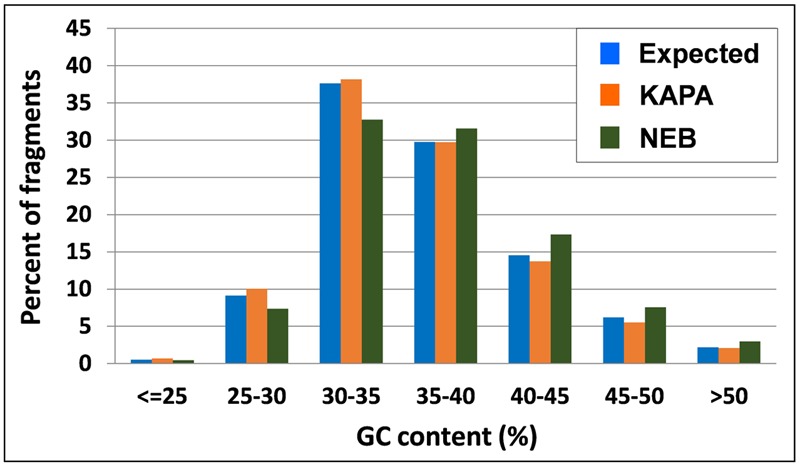
**Tag distribution across different GC contents of DNA fragments in *M. truncatula* as expected from *Ape*KI *in silico* digestion and observed from 2 *Taq* polymerases**.

The inspection of observed vs. expected frequencies for tag classes that differ for relative content of GC nucleotides revealed more homogeneous amplification by KAPA also across different GC contents. In particular, NEB tended to overamplify the fragments whose GC content exceeded 35%, while underamplifying those with GC content below this level (**Figure 3**).

### Comparison of *Taq* Polymerase by Selective Primer Combinations in Pea, White Lupin, and Alfalfa

**FIGURE 4:**
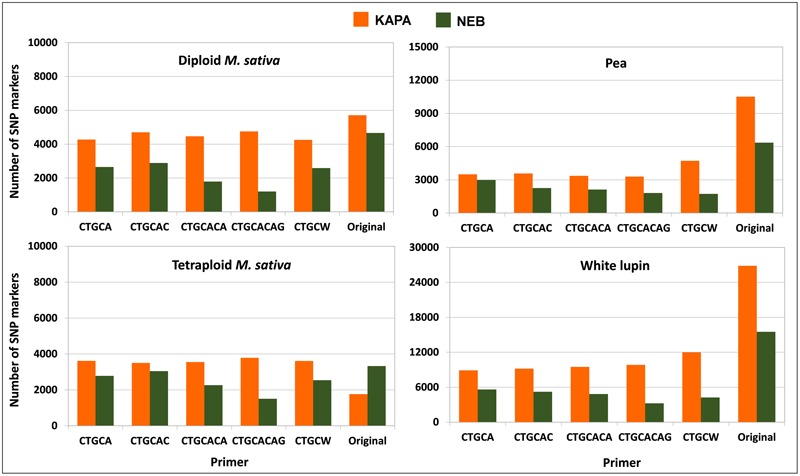
**Number of polymorphic SNP markers shared by 6 pea genotypes, 4 white lupin genotypes, 4 genotypes of diploid *M. sativa* and 2 of tetraploid *M. sativa*, for 2 *Taq* polymerase by 6 primer combinations.** Minimum read depths of 3 for pea and white lupin, 6 for diploid *M. sativa*, and 11 for tetraploid *M. sativa*; 1.5 M total reads per genotype.

The protocol combining KAPA polymerase with the non-selective primer (original method) outperformed any other protocol in terms of number of polymorphic SNP loci for white lupin, pea, and diploid alfalfa (**Figure 4**). The advantage of this protocol was very large in white lupin and large in pea, in coincidence with the low minimum read depth required for SNP calling in these inbred species. The advantage was more limited but still sizeable for diploid alfalfa, where it agreed with earlier results for the KAPA vs. NEB comparison in the presence of the non-selective primer that are reported in **Figure 1B** for the same minimum read depth of 6 and the scenario of 2.5 M total reads per genotype. However, KAPA combined with a selective primer (with minor differences among such primers), or NEB without a selective primer, provided more polymorphic loci than KAPA without a selective primer for the outbred tetraploid *M. sativa* (which requires higher minimum read depth) (**Figure 4**). In all species, the adoption of a selective primer was more beneficial in combination with KAPA than NEB (**Figure 4**).

**FIGURE 5:**
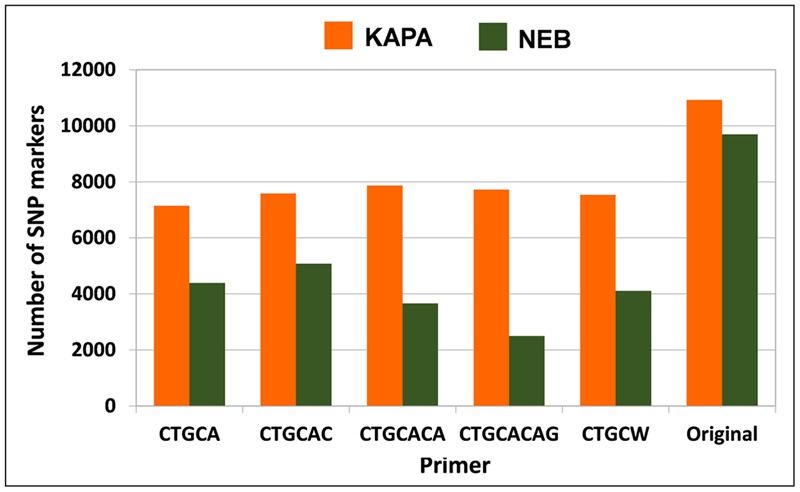
**Number of polymorphic SNP markers shared by 2 genotypes of tetraploid *M. sativa*, for 2 *Taq* polymerase by 6 primer combinations.** Minimum read depth of 11; 2.5 M total reads per genotype.

The analysis for the scenario of 2.5 M total reads per genotype, which was performed only for tetraploid alfalfa, indicated a slight advantage of the protocol including KAPA with the non-selective primer (**Figure 5**), in contrast with results for the scenario of 1.5 M total reads per genotype (**Figure 4**). Using NEB with the non-selective primer was nearly as good, however (**Figure 5**).

### Number of Polymorphic Markers and Predictive Ability of Genomic Selection Models in Different Data Sets

Genotyping-by-sequencing has been used to genotype several forage or cool-season grain legumes (**Table 1**). Most reported data sets have been sequenced with approximately 96 samples per lane (in some cases, other samples than those listed in the table were included in a lane in order to generate a 96-plex). All grain legume data sets including a RIL population displayed over 2,300 polymorphic SNP markers, with the exception of one chick pea data set whose lower number of markers may partly be due to more stringent filtering criteria that were adopted for SNP calling or possibly a narrower genetic diversity between the parents of the RILs. In white lupin, a world collection of landraces displayed over 2.6-fold more markers than a RIL population, as expected from the greater genetic diversity of this type of germplasm set. Finally, the number of polymorphic SNP markers exceeded 6,500 in all data sets of tetraploid alfalfa.

**Table 1:** Mean number of total reads per genotype, and number of polymorphic single nucleotide polymorphism (SNP) markers, in different data sets.

Germplasm set	No. of genotypes	No. of reads/genotype (M)	No. of SNPs	Source
Tetraploid alfalfa, Medit. material^a^	154	2.89	10,339	Annicchiarico et al., 2015b
Tetraploid alfalfa, Po Valley material^a^	124	2.75	6,690	Annicchiarico et al., 2015b
Tetraploid alfalfa, US material^b^	190	2.36	9,906	Li et al., 2015
Pea, Attika × Isard lines^c^	105	2.40	2,386	Annicchiarico et al., 2017
Pea, Kaspa × Attika lines^c^	105	1.85	2,506	Annicchiarico et al., 2017
Pea, Kaspa × Isard lines^c^	105	2.30	2,750	Annicchiarico et al., 2017
White lupin, Kiev × P27255 lines^c^	191	1.90	2,593	Unpublished data
White lupin, world landrace pool^c^	288	1.66	6,802	Unpublished data
Chickpea, SBD377 × BGD112 lines^d^	95	1.80	3,977	Verma et al., 2015
Chickpea, ICC4958 × ICC1882 lines^e^	210	3.37	828	Jaganathan et al., 2015

*^a^Minimum no. of reads: 4/11 for heterozygous/homozygous loci; genotype missing data threshold: 30%.*

*^b^Minimum no. of reads: 2/11 for heterozygous/homozygous loci; genotype missing data threshold: 35%.*

*^c^Minimum no. of reads: 4; genotype missing data threshold: 30%.*

*^d^Minimum Qscore of 10 for read retention; unspecified genotype missing data threshold.*

*^e^Minimum Qscore of 20 for read retention; genotype missing data threshold: 50%.*

No comparison between GBS and SNP array procedures for number of polymorphic SNP markers is available for these or other legume data sets. However, the three pairs of parent genotypes that originated the RIL populations of pea displayed, on average, 3,925 polymorphic SNP markers according to the SNP array facility described by Tayeh et al. (2015a) (Grégoire Aubert and Judith Burstin, pers. comm.). In comparison, the GBS-generated polymorphic SNP markers in the three RIL populations originated by these parents amounted, on average, to 2,547 for the genotype missing data threshold of 30% (**Table 1**), and 4,409 for the missing data threshold of 50%.

**FIGURE 6:**
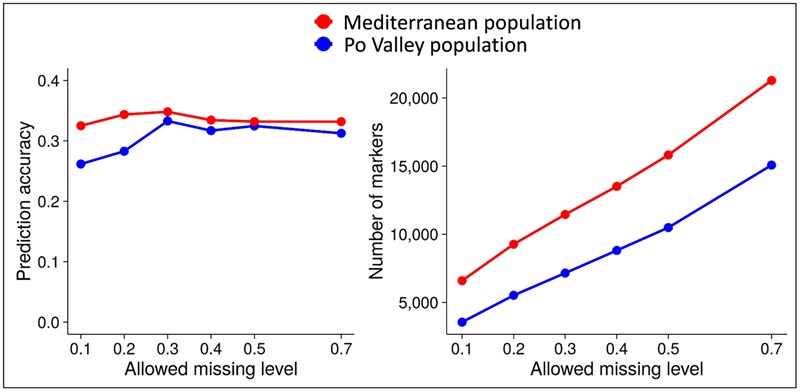
**Predictive ability of genomic selection for forage yield and number of polymorphic SNP markers as a function of different genotype missing data thresholds, in two data sets of tetraploid alfalfa.** Source of data: Annicchiarico et al. (2015b).

In earlier work of ours on alfalfa and pea (Annicchiarico et al., 2015b, 2017; Biazzi et al., 2017), the predictive ability of genomic selection was affected by the threshold for genotype SNP missing data, the method for imputing missing data, and the genomic selection model. Increasingly relaxed missing data threshold, while increasing the number of polymorphic markers, displayed a peak of predictive ability in the range of 20–40% missing data. This is reported in **Figure 6** for forage yield of alfalfa in two data sets, and in Annicchiarico et al. (2017) for grain yield of pea RILs (for minimum read depth of 4 or 6). For missing data imputation, we found an advantage of Random Forest imputation over other methods based on Singular Value Decomposition, Localized Haplotype Clustering, or Mean imputation (Annicchiarico et al., 2015b). However, *K*-Nearest Neighbors imputation proved about as reliable as Random Forest, while being much faster computationally (Nazzicari et al., 2016).

The analysis of various legume data sets indicated that the predictive ability of genomic selection can be affected by the genomic selection model, but no model proved unanimously optimal across different traits or data sets (although in most cases, the differences among models were not large). For example, Support Vector Regression with linear kernel outperformed Bayes A, Bayes B, and Bayesian Lasso models for predicting alfalfa forage yield in two data sets (Annicchiarico et al., 2015b), while tending to be outperformed by Bayesian methods (especially Bayesian Lasso) for prediction of pea grain yield (Annicchiarico et al., 2017) and various leaf and stem quality traits of alfalfa (Biazzi et al., 2017). Ridge Regression BLUP (or a model analogous to it, e.g., GBLUP) tended to be among the most accurate models in all of these studies, as well as in two pea studies based on Infinium array SNP markers (Burstin et al., 2015; Tayeh et al., 2015b). In the current comparison of five genomic selection models for two alfalfa quality traits, Ridge regression BLUP and Bayes C revealed a slight advantage for crude protein content and NDF digestibility, respectively, in the absence of marked differences in predicting ability among all tested models (Supplementary Figure 1).

Predictive ability values of the best-performing genomic selection models for yield or key quality traits of alfalfa or pea using GBS-generated SNP data ranged up to 0.72 (**Table 2**). Values for alfalfa breeding values ranged between 0.31 and 0.36. Results for pea grain yield were averages of 3 RIL populations reported in **Table 1**. They were higher than those for alfalfa breeding values for biomass yield, ranging from 0.72 under growing conditions experiencing severe terminal drought to 0.48 for northern Italy under autumn sowing (**Table 2**). Chick pea results were not available, since data sets in **Table 1** were used for GWAS.

**Table 2:** Predictive ability of genomic selection for genotype breeding value in different data sets.

Germplasm set	Trait	Predictive ability	Source
Tetraploid alfalfa, Medit. material^a^	Biomass yield	0.36	Annicchiarico et al., 2015b
Tetraploid alfalfa, Po Valley material^a^	Biomass yield	0.32	Annicchiarico et al., 2015b
Tetraploid alfalfa, US material^b^	Biomass yield	0.31	Li et al., 2015
Alfalfa, Medit. germplasm^a^	NDF digestibility	0.32	Unpublished data
Alfalfa, Medit. germplasm^a^	Crude protein content	0.32	Unpublished data
Pea, average of three connected crosses^c^	Grain yield, severe drought	0.72	Annicchiarico et al., 2017
Pea, average of three connected crosses^c^	Grain yield, north Italy	0.48	Unpublished data

*^a^Minimum no. of reads: 4/11 for heterozygous/homozygous loci; genotype missing data threshold: 30%.*

*^b^Minimum no. of reads: 2/11 for heterozygous/homozygous loci; genotype missing data threshold: 35%.*

*^c^Minimum no. of reads: 4; genotype missing data threshold: 30%.*

### Comparison of Genomic vs. Phenotypic Selection Scenarios

With the exception of Li et al. (2015), the predictive ability values reported in **Table 2** relate to predictions for the same test environment using cross-validations. Predictions for other test environments (as in the ordinary use of genomic selection) are bound to be less accurate, owing to genotype-by-environment (GE) interactions between the environment(s) used for model definition and those used for application of the model. This is especially true for crop yield, which is usually exposed to wider GE interaction than quality traits. However, the key issue in relation to GE interaction for both genomic and phenotypic selection is the ability to predict the genotype breeding values for the target environments of the breeding program. In Li et al. (2015), genomic selection models from NY and Québec were good at predicting each other’s phenotyping data, as may be expected from the geographic proximity of their test sites.

Cross-environment predictions can be incorporated into formulas for predicting yield gains from one cycle of phenotypic or genomic selection within a given genetic base. For outbred species such as alfalfa, the predicted gain per year from phenotypic selection (Δ*G*_P_) is:

$$\Delta G_{P}\;=\;(i_{P}hs_{A}r_{g}{}_{P})/t_{P}$$

where *i*_P_ is the standardized selection differential, *h* is the square root of narrow-sense heritability in the selection conditions, *s*_A_ is the standard deviation of breeding values, *r*_gP_ is the genetic correlation for genotype yield responses between selection and target conditions, and *t*_P_ is the number of years for one phenotypic selection cycle. The predicted gain from genomic selection (Δ*G*_G_) is:

$$\Delta G_{G}\;=\;(i_{G}r_{A}s_{A}r_{gG})/t_{G}$$

where *i*_G_ is the standardized selection differential for genomic selection, *r*_A_ is the genomic selection accuracy, *r*_gG_ is the genetic correlation for genotype yield responses between phenotyping conditions for genomic selection modeling and target conditions, and *t*_G_ is the duration of one genomic selection cycle. Assuming the same testing conditions (*r*_gP_ = *r*_gG_) and selection intensities (*i*_P_ = *i*_G_), a comparison of phenotypic vs. genomic selection in terms of predicted yield gain per year equates to comparing (*h*/*t*_P_) vs. (*r*_A_/*t*_G_). For the alfalfa reference population from the Po Valley, the estimated values of *r*_A_ = 0.32 (**Table 1**) and *h* = 0.46 [from *h*^2^ = 0.21 in Annicchiarico (2015)] suggest that genomic selection would result in higher gain than phenotypic selection if it could halve the duration of one selection cycle. Actually, considering that *t*_G_ = 1, and *t*_P_ = 5 (**Table 3**) when including the time for recombination of selected material and for phenotypic selection along with 1 year for prior production of half-sib families, this criterion was easily met. Even *r*_A_ = 0.15 would suffice to grant some advantage to genomic selection over half-sib progeny-based phenotypic selection according to this criterion.

**Table 3:** Duration of one selection cycle and indicative cost per evaluated genotype, for hypothetical scenarios of phenotypic and genomic selection for higher yield.

Selection	Selection cycle (years)	Cost per genotype (€)^a^
*Cool-season grain legume*		
Phenotypic (1 site, 2 years, 3 reps)	2	180–230
Genomic	0.5	32–36
Ratio phenotypic/genomic	4	5.0–7.2
*Perennial forage legume*		
Progeny test (1 site, 3 years, 3 reps)	5	230–280
Genomic	1	32–36
Ratio phenotypic/genomic	5	6.4–8.7

*^a^Excluding bioinformatics/data analysis work.*

*Grain legumes: selection of inbred lines; forage legumes: selection of parents for a synthetic variety.*

For inbred species, the reported formulas for estimating expected genetic gains hold true, when *h* is substituted for by the square root of broad-sense heritability on an entry mean basis (*H*) under the specific conditions adopted for phenotypic selection:

$$H^{2}\;=\;s_{g}{}^{2}/(s_{g}{}^{2}\;+\;s_{ge}{}^{2}/e\;+\;s_{e}{}^{2}/er)$$

where *s*_g_^2^, *s*_ge_^2^ and *s*_e_^2^ are components of variance relative to genotype, GE interaction and pooled experiment error, and *e* and *r* are the numbers of test environments and experiment replications, respectively. Extensive multi-environment phenotypic selection (high *e* values) could rise *H* near unity, but this is usually prevented by its high cost.

The comparison of selection methods for predicted yield gain could incorporate the evaluation cost per genotype. For example, even the limited multi-environment phenotypic selection scenarios hypothesized in **Table 3** result, on average, in about 6-fold greater cost for grain legumes and 7.5-fold greater cost for forage legumes of phenotypic selection relative to genomic selection. Thus, for same total evaluation cost of the two methods, more genotypes could be evaluated by GBS, increasing the selection intensity (*i*_G_). For alfalfa, a comparison of phenotypic vs. genomic selection in terms of predicted yield gains per year for the same overall costs equates to comparing (*h i*_P_/*t*_P_) vs. (*r*_A_ *i*_G_/*t*_G_). For example, the phenotypic selection based on progeny-testing of 300 alfalfa genotypes aimed to select 15 parents for a synthetic variety (selected fraction = 5%) implies *i*_P_ = 2.06 (Falconer, 1989), whereas the genomic selection based on evaluating 2250 genotypes (7.5-fold more than phenotypic selection) that aimed to select 15 parents (selected fraction = 0.66%) implies *i*_G_ = 2.80. For the alfalfa reference population from the Po Valley and the selection scenarios hypothesized in **Table 3**, genomic selection leads to over 4.7-fold greater predicted yield gain per year than phenotypic selection (from 0.46 × 2.06/5 = 0.189 for phenotypic selection vs. 0.32 × 2.80/1 = 0.896 for genomic selection). From this perspective, *r*_A_ = 0.15 (which implies over twofold greater predicted gains for genome-enabled selection) would justify the inclusion of genomic selection in breeding schemes, for the cost and selection cycle scenarios reported in **Table 3**.

For pea in Italian environments, assuming for example the selection of 15 genotypes phenotypically out of 300 or genomically out of sixfold more test genotypes (hence, *i*_P_ = 2.06, and *i*_G_ = 2.39) under the scenario in **Table 3** (*t*_P_ = 2; *t*_G_ = 0.5), and considering *r*_A_ = 0.48 (i.e., the lower value for pea in **Table 2**) and a cautiously high estimate of *H* = 0.84 that arises from a multi-environment study in Italy by Annicchiarico and Iannucci (2008) with *e* = 7 (rather than *e* = 4 as in **Table 3**), would result in 2.6-fold greater predicted yield gain per year of genomic selection relative to phenotypic selection. The comparison of phenotypic vs. genomic selection equates here to comparing (*H i*_P_/*t_P_*) vs. (*r*_A_ *i*_G_/*t*_G_) (i.e., 0.84 × 2.06/2 = 0.865 for phenotypic selection vs. 0.48 × 2.39/0.5 = 2.294 for genomic selection). An *r*_A_ = 0.36 would provide a twofold advantage for genomic selection under these circumstances.

## Discussion

Our results relative to comparisons of major components of GBS protocols (REs, *Taq* polymerases, primers) cannot be considered conclusive, but they indicated that each of these components may have a large effect on the number of SNP markers generated by GBS. Also, they highlighted the importance of investigating combinations of these components (such as different *Taq* polymerases in combination with different primers or REs), because results for each individual component may vary depending on other components of the GBS protocol.

Reducing the number of target sites through DNA digestion by *Pst*I:*Msp*I instead of *ApeK*I was not advantageous for diploid alfalfa or *M. truncatula* at convenient read depths for these species. Results for diploid alfalfa provided indirect evidence for the greater interest of *ApeK*I over *Pst*I:*Msp*I even for tetraploid alfalfa at 2.5 M total reads per genotype, which is the ordinary scenario for data sets of this crop (**Table 1**). A contributing reason for the advantage obtained from greater genome complexity reduction by using *Pst*I:*Msp*I instead of *ApeK*I in Poland et al. (2012a) could be the larger genome of their target species relative to alfalfa (about 19- and 6-fold larger estimated genome for wheat and barley, respectively). In addition, the current adoption of KAPA polymerase could amplify the advantage of *ApeK*I over *Pst*I:*Msp*I in comparison with NEB polymerase, which was used in Poland et al.’s (2012a) study. *ApeK*I proved preferable to two less frequent-cutting enzymes also for cassava, whose genome size is comparable to alfalfa (Hamblin and Rabbi, 2015).

Selective primers displayed an advantage only in the presence of high minimum read depth and low sequencing effort. In alfalfa (requiring 11 as minimum read depth), selective primers proved advantageous at 1.5 M total reads per genotype but not at 2.5 M total reads. Indeed, one may expect greater advantage from greater mean read depth per SNP obtained via reduction of target sites when adopting low sequencing levels. The advantage of the non-selective primer emerged already at 1.5 M total reads per genotype in diploid alfalfa (minimum read depth of 6), and was particularly large for pea and white lupin (minimum read depth of 2). Our results contrast with those by Sonah et al. (2013) for soybean holding 2 as minimum read depth, where selective primers increased the number of polymorphic SNP markers under scenarios of 1–2 M total reads per genotype. This inconsistency encourages further investigations, also in consideration of the small set of genotypes that provided the basis for the polymorphic SNP assessment in these studies [6 to 2 genotypes here; 2 genotypes in Sonah et al. (2013)].

Results for number of tags or polymorphic markers indicated that KAPA can be preferred to NEB *Taq* polymerase for diploid alfalfa, pea, and white lupin. It is preferable also for tetraploid alfalfa in the ordinary scenario of 2.5 M total reads per genotype. Additionally, our results for *M. truncatula* indicated more uniform amplification over fragment sizes and GC contents of this polymerase relative to NEB. This finding has clear potential for improving GBS-based activities on legumes, since most GBS protocols use NEB. Further comparisons are warranted, however, for other forage or grain legume species.

We assumed a minimum read depth of 2 for pea and white lupin in our assessment of GBS protocols. However, a higher value, such as 4, could conveniently be set for lines that may include some degree of heterozygosity, as we did for the pea RILs that underwent our genomic selection assessment. Anyway, best *Taq* polymerase × selective primer combinations did not change for these species when considering a minimum read depth of 4 instead of 2 (data not reported).

On the whole, our results support the adoption of a single successful GBS protocol for tetraploid and diploid alfalfa, pea, and white lupin, using *ApeK*I and the non-selective primer as in the original method (Elshire et al., 2011) along with KAPA polymerase. The good overall performance of this protocol might serve as a reference for GBS work in other forage or cool-season legumes that lacks an experimental assessment of protocol components. The value of this protocol ought to be reassessed for tetraploid alfalfa if accurate allele dosage information was desired, which would require about 48x read depth to differentiate the heterozygote classes (Uitdewilligen et al., 2013). We did not consider this scenario, because the large sequencing effort required to obtain thousands of markers with sufficient read depth is currently prevented by its high cost.

We are mainly interested in using GBS for genomic selection to improve crop yield, a complex, highly polygenic trait. No statistical model consistently maximized the predictive ability across different data sets, confirming the scope for exploring different models in genomic selection studies. Ridge Regression BLUP (or its analog GBLUP) ought to be included among the tested models in all cases, on the basis of its currently good performance in different situations and its theoretical suitability for a trait controlled by many loci with small effects (as crop yield is expected to be) (Lorenz et al., 2011). Bayesian Lasso proved to be another well-performing model in most of our analyses. Indeed, these models proved well-performing across a range of plant and animal data sets (de los Campos et al., 2013).

There are two other suggestions for data analysis that descend from our experience. One is imputing missing data by Random Forest [in agreement with results for other species: e.g., Poland et al. (2012b)] or by *K*-Nearest Neighbors, until a sequenced genome will allow for using other methods, e.g., Beagle (Nazzicari et al., 2016). The other is the need for assessing the predictive ability across a range of missing data thresholds. The peak of predictive ability that we found between 20 and 40% missing data, which agrees with other results for soybean (Jarquín et al., 2014) and most results for alfalfa reported by Li et al. (2015), is consistent with the expected trade-off between increased information (more markers) and increased noise (higher imputation errors) that arises from increasing threshold for missing data.

Genotyping-by-sequencing can be used to produce high numbers of polymorphic SNP markers for forage and cool-season grain legumes. For three pea RIL populations, the number of polymorphic markers generated by GBS was comparable to that expected at much higher cost by a SNP array genotyping. Particularly when used with best model configurations, GBS-based genomic predictions were sufficiently high for cost-effective exploitation by breeding programs. The occurrence of more accurate predictions in pea than alfalfa could be expected, owing to the much longer linkage disequilibrium and the possibility to thoroughly exploit also non-additive genetic variation that feature the RILs of an inbred crop compared with a set of progeny-tested alfalfa parents. Genomic selection accuracy for soybean grain yield based on cross-validations, which achieved 0.64 (Jarquín et al., 2014), is intermediate between the values of 0.72 and 0.48 that we found for pea grain yield. The difference in prediction accuracy between the two data sets of pea could be attributed to the different ecological complexity of the two phenotyping environments. The environment prone to severe terminal drought, which reproduced a climatically unfavorable Mediterranean environment, was ecologically simpler, because higher genotype yield was strictly associated genetically with an early phenology (Annicchiarico et al., 2017). Higher yield in the autumn-sown, subcontinental-climate environment of northern Italy required genotype adaptation to both low winter temperatures and terminal drought.

We proposed a simple general framework for comparing genomic vs. phenotypic selection in terms of expected genetic gain, which takes account of differences in selection cycle duration and genotype evaluation cost between the selection methods. These differences can be substantial, and their impact on the relative efficiency of selection methods can be important. We chose only one example scenario among many possible ones for comparing genomic vs. phenotypic selection in each crop, and we lacked estimates for important parameters such as *r*_gP_ and *r*_gG_. In the absence of these estimates, the ability of genomic and phenotypic selection to predict genotype yields in cropping environments other than the test one could be highly informative, but also this information was not available. Though limited, our preliminary comparison revealed a large predicted advantage of genomic selection over phenotypic selection that is encouraging for legume breeding and supports further and more conclusive assessments of genome-enabled predicting ability across a wider set of cropping environments.

Rajsic et al. (2016) proposed another method for comparing genomic vs. phenotypic selection in inbred crops that accounts for different selection costs, in which genomic prediction accuracy is estimated as a function of trait heritability, effective number of chromosome segments underlying the trait, and training population size. When setting *H*^2^ = 0.85 for pea, already a twofold cost of phenotypic selection relative to genomic selection would imply some predicted advantage for genomic selection across a wide range of effective number of chromosome segments. With no account for different selection costs or selection cycle duration, simulation results by Viana et al. (2016) for outbred species suggest greater predicted yield gain per selection cycle by genomic selection when *h^2^* is below 0.30 (as here for alfalfa), for a scenario of 200 genotyped individuals and moderate sequencing effort.

Another issue of interest is the ability of a model set up for a given genetic base to predict the same trait in a different genetic base. Cross-population predictions for alfalfa biomass of Mediterranean germplasm based on a model from Po Valley germplasm or vice versa implied just a moderate loss of predictive ability (25–30%) relative to intra-population predictions (Annicchiarico et al., 2015b). Preliminary results for pea revealed small to quite large loss of predictive ability passing from intra-population to cross-population prediction of grain yield, depending on the pair of RIL populations (Annicchiarico et al., 2017).

We valued genomic selection mainly for its ability to increase the rate of genetic yield gain through shorter selection cycles and more evaluated genotypes for same overall cost. Another contribution of genomic selection to crop improvement could be its unprecedented potential for selecting simultaneously for several traits. This can be particularly important for perennial forage legume breeding, which is constrained by high phenotyping costs and requires at least 10–15 selected genotypes as parents of a synthetic variety (Annicchiarico et al., 2016). For example, selecting 10 genotypes for 4 traits at the modest selection rate of 20% for each trait requires a working population of [10 × (1/0.20)^4^] = 6,250 individuals, a number that is hardly workable for phenotypic selection in these crops (particularly when involving a time- and resource-consuming trait, such as forage yield across several harvests and production cycles) while being within reach for genomic selection (particularly in the perspective of continuously decreasing genotyping costs). The moderately high genome-enabled predictive ability that emerged for two important alfalfa forage quality traits, namely, digestibility of NDF and protein content, has practical interest and supports this perspective use of genomic selection. While higher protein content is beneficial to decrease the dependency of crop-livestock systems from expensive extra-farm feed protein sources, higher NDF digestibility is the main determinant of cattle dry-matter intake and milk yield (Oba and Allen, 1999).

We expect a steep rise in genomic selection studies in forage and cool-season grain legumes in the next few years, especially because of the promising results that have emerged from the first studies (such as those reported here). GBS data will probably be pivotal in this context, owing to their low cost and possible usefulness also for identifying candidate genes in GWAS as soon as a sequenced genome becomes available for these crops. Challenges arising from GBS-based genotyping (adopted protocol; SNP calling procedure; method of missing data imputation; etc.) have not been trivial in pioneering work, but are bound to be overcome by increasing scientific knowledge, availability of sequencing platforms and development of bioinformatic tools.

## Author Contributions

PA and EB designed and supervised the research work, and obtained financial resources. NN was responsible for bioinformatics analysis. YW was responsible for lab experiments and generation of molecular data. LP and PA were responsible for phenotyping experiments. PA drafted the manuscript. All authors revised, integrated, and approved the manuscript.

## Conflict of Interest Statement

The authors declare that the research was conducted in the absence of any commercial or financial relationships that could be construed as a potential conflict of interest.

## Footnotes

1 http://homes.cs.washington.edu/~dcjones/fastq-tools/
