# Supplementary figures and images for: Genotyping-by-Sequencing and Its Exploitation for Forage and Cool-Season Grain Legume Breeding
Source: Front Plant Sci. 2017 May 9;8:679. doi: 10.3389/fpls.2017.00679 (PMC5423274; doi:10.3389/fpls.2017.00679)

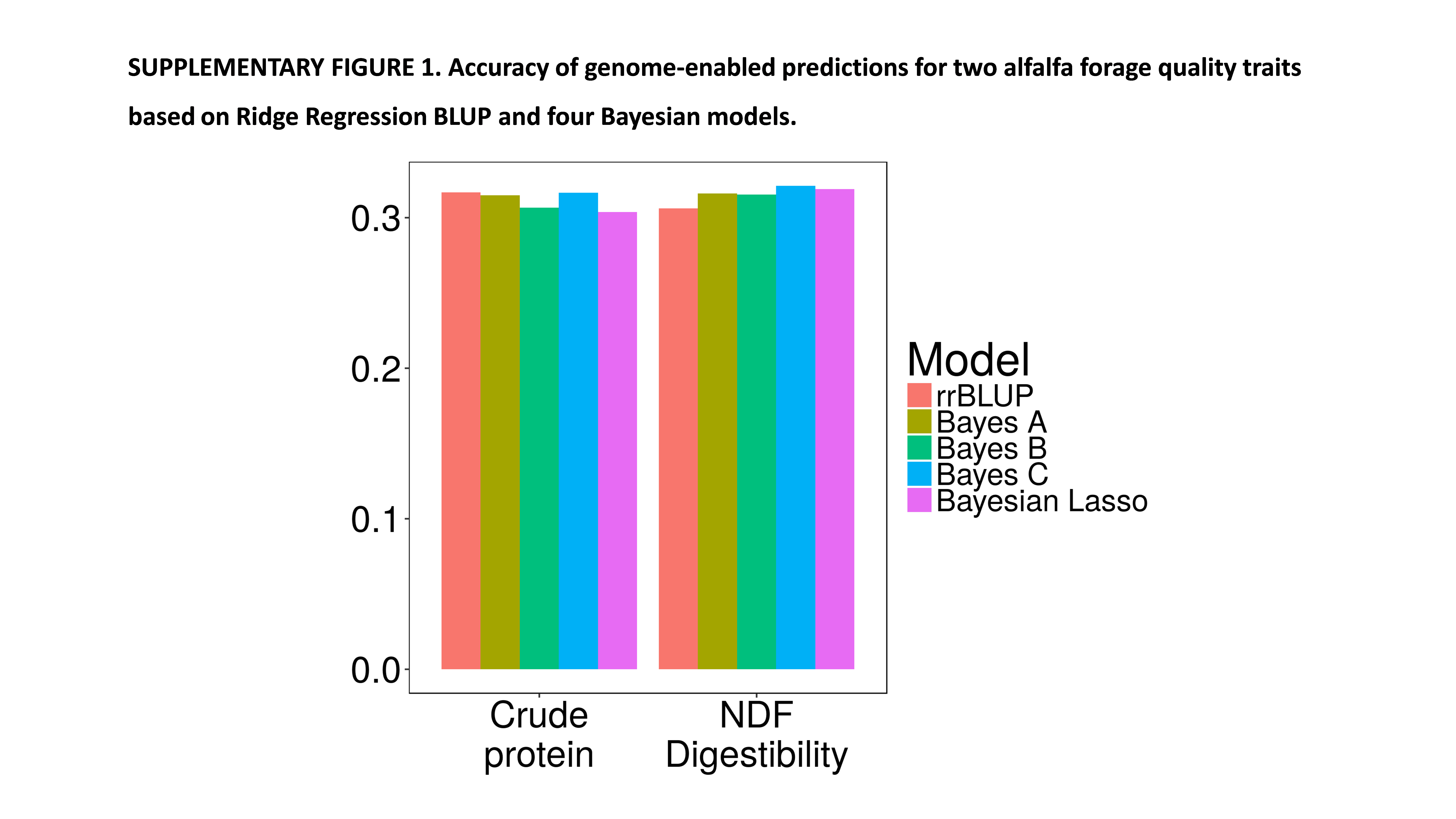

Supplement: Supplementary file 1 [file Image_1.tif]
